# In Vitro Bile Salt Hydrolase (BSH) Activity Screening of Different Probiotic Microorganisms

**DOI:** 10.3390/foods10030674

**Published:** 2021-03-22

**Authors:** Jimmy G. Hernández-Gómez, Argelia López-Bonilla, Gabriela Trejo-Tapia, Sandra V. Ávila-Reyes, Antonio R. Jiménez-Aparicio, Humberto Hernández-Sánchez

**Affiliations:** 1 Departamento de Ingeniería Bioquímica, Escuela Nacional de Ciencias Biológicas, Instituto Politécnico Nacional, Av. Wilfrido Massieu 399, UP Adolfo López Mateos, Mexico City CP 07738, Mexico; jimmyherdz@gmail.com; 2 Centro de Desarrollo de Productos Bióticos, Instituto Politécnico Nacional, Yautepec CP 62731, Mexico; alopezb@ipn.mx (A.L.-B.); gttapia@ipn.mx (G.T.-T.); sandra_victory@yahoo.com (S.V.Á.-R.); arjaparicio@gmail.com (A.R.J.-A.)

**Keywords:** bile salt hydrolase, probiotics, *Lactobacillus plantarum*, *Saccharomyces boulardii*, cholesterol

## Abstract

Bile salt hydrolase (BSH) activity in probiotic strains is usually correlated with the ability to lower serum cholesterol levels in hypercholesterolemic patients. The objective of this study was the evaluation of BSH in five probiotic strains of lactic acid bacteria (LAB) and a probiotic yeast. The activity was assessed using a qualitative direct plate test and a quantitative high-performance thin- layer chromatography assay. The six strains differed in their BSH substrate preference and activity. *Lactobacillus plantarum* DGIA1, a potentially probiotic strain isolated from a double cream cheese from Chiapas, Mexico, showed excellent deconjugation activities in the four tested bile acids (69, 100, 81, and 92% for sodium glycocholate, glycodeoxycholate, taurocholate, and taurodeoxycholate, respectively). In the case of the commercial probiotic yeast *Saccharomyces boulardii*, the deconjugation activities were good against sodium glycodeoxycholate, taurocholate, and taurodeoxycholate (100, 57, and 63%, respectively). These last two results are part of the novelty of the work. A weak deconjugative activity (5%) was observed in the case of sodium glycocholate. This is the first time that the BSH activity has been detected in this yeast.

## 1. Introduction

Cardiovascular diseases are the leading cause of mortality in the world among adults between 35 and 70 years old, with more than 18 million deaths in 2019. They are classified as civilization diseases, so preventing them is very important [1]. In modern societies, ingesting high-fat, high-cholesterol diets usually leads to hypercholesterolemia and atherosclerosis [2]. The World Health Organization (WHO) has warned that by the year 2030, ischemic heart disease will continue to be the leading cause of death worldwide, with a death toll of around 23.6 million people. Hypercholesterolemic patients may avoid the use of cholesterol-lowering drugs such as statins [3], through diet control, exercise, weight loss, and supplementation with probiotics [4].

Probiotics, in particular, lactic acid bacteria (LAB), are live microorganisms that have beneficial health effects when administered in the right amounts. Eating them with everyday food (cheese, fermented milks, etc.) can prevent many diseases, including hypercholesterolemia [5]. Several mechanisms for cholesterol removal by probiotics have been shown, such as deconjugation of bile salts by the enzyme bile salt hydrolase (BSH), the production of short-chain fatty acids, the assimilation of cholesterol into bacterial cell membranes, and the conversion of cholesterol by hydrogenation to the poorly absorbed sterol coprostanol [6]. 

**Figure 1:**
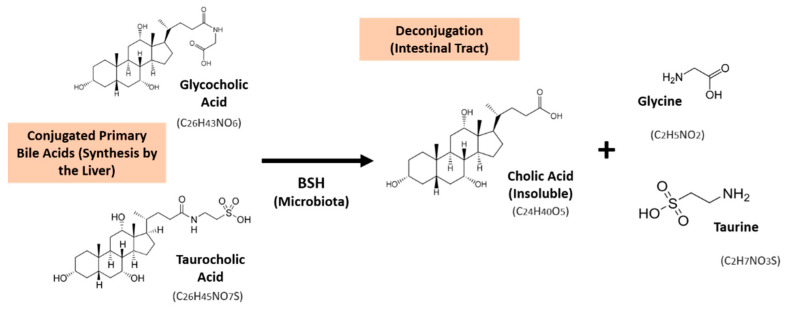
Deconjugation reaction of the glycocholic and taurocholic acids by the microbial bile salt hydrolase (BSH) present in the intestinal tract. A similar reaction occurs for the deconjugation of the secondary bile salts (glycodeoxycholic and taurodeoxycholic acids).

Bile acids are produced from cholesterol inside the liver. Cholic acids belong to the primary bile acids class and are synthesized by hepatocytes, then excreted into the intestinal tract as glycine or taurine conjugates. In turn, primary bile acids can be used as substrates for bacterial biotransformation to secondary bile acids in the colon. Many probiotic microorganisms have the ability to produce the enzyme BSH, which catalyzes the deconjugation of bile salts linked with glycine or taurine, as shown in Figure 1 [7]. 

This characteristic has evolved as a mechanism to tolerate the antimicrobial effect of conjugated bile acids in the small intestine [8]. These unconjugated bile acids are eventually excreted in the feces, having an overall effect of lowering the serum cholesterol level in people with hypercholesterolemia [9,10]. Glycine or taurine are released when the amide bonds undergo hydrolysis by the BSH, and can be used as carbon sources by the intestinal microbiota. It has been shown that bacteria belonging to the genera *Lactobacillus, Bifidobacterium*, and *Enterococcus* possess BSH activity [9]. For adequate activity, this enzyme must be delivered in the proximal small intestine [11]. However, the exact site of release using live bacteria is less predictable than other systems, such as microencapsulated BSH. Nevertheless, the cost of using purified enzymes in microencapsulation is an important issue, even in the case of crude enzyme extracts [9]. A possible solution usually involves finding a wild strain that is able to produce high amounts of the enzyme or a GMO in which the *bsh* gene could be cloned and expressed for overproduction of the BSH [12]. The following research was focused on the quantitative screening of the most promising probiotic strains with superior in vitro bile-salt-hydrolyzing capacity, so supplements or foods that include them could be recommended for daily consumption for the lowering of serum cholesterol.

## 2. Materials and Methods

### 2.1. Microorganisms

Five lactic acid bacteria (LAB) strains of the genus *Lactobacillus* and a yeast were used in this work: *Lactobacillus casei* Shirota was isolated from the fermented dairy beverage Yakult^®^. *L. plantarum* 299v was isolated from capsules of the supplement Protransitus LP^®^. *L. rhamnosus* GG was isolated from packets of the supplement Vivera^®^. *L. plantarum* DGIA1 was isolated from a double cream cheese from the Mexican State of Chiapas [13]. *L. fermentum* K73 was isolated from Colombian suero costeño (traditional fermented sour cream) [14]. *Saccharomyces cerevisiae* var. *boulardii* CNCM I-745 was isolated from capsules of the supplement Floratil^®^ [15]. The LAB were kept frozen at −70 °C in MRS broth (YPD broth for the yeast) supplemented with 50% glycerol (*v*/*v*) until used.

### 2.2. Qualitative Direct Plate BSH Assay

In the case of the LAB strains, sterile filter discs (6 mm) were saturated with an overnight culture in MRS broth and then placed on MRS agar plates supplemented with 0.5% (*w*/*v*) sodium salt of glycocholic, glycodeoxycholic, taurocholic, or taurodeoxycholic acids (Sigma-Aldrich, St. Louis, MO, USA) and 0.37 g/L CaCl_2_. The plates were incubated at 37 °C for 72 h, after which the diameter of the deconjugated bile acid precipitation zones (opaque halos) was measured. In the case of the yeast *S. boulardii*, the procedure was very similar, but YPD broth and agar were used instead of MRS. MRS or YPD agar medium plates without supplementation of the conjugated bile acids were used as controls [6,10].

### 2.3. Quantitative High-Performance Thin-Layer Chromatography (HPTLC) BSH Assay

To determine the BSH activity, the quantitative HPTLC method described by Rohawi et al. [16] was used with some modifications. Briefly, the probiotic microorganisms were cultured at 37 °C for 24 h in tubes with MRS broth (YPD broth in the case of *S. boulardii*) containing 1 mmol/L of the sodium salts of glycocholic, glycodeoxycholic, taurocholic, or taurodeoxycholic acids. Uninoculated tubes were used as controls. The cultures were centrifuged at 1300× *g* at 4 °C for 10 min, and the cell-free supernatant was aliquoted and stored at −80 °C until use.

The HPTLC plates (20 × 10 cm^2^ with silica gel 60 with fluorescent indicator F254) were oven-activated at 105 °C for 15 min and cooled in a desiccator. Samples were applied to the plates as 8 mm bands using a Linomat 5 with a 25 µL syringe using nitrogen as the propellant (Camag^®^, Muttenz, Switzerland). The bands were 10 mm apart and 15 mm from the lower edge of the plates, and 17 lanes were used in each plate. Stock standard solutions of the sodium salts of glycocholic, glycodeoxycholic, taurocholic, or taurodeoxycholic acids were prepared in methanol (1 µg/mL). From the stock standard solution, volumes from 1 to 12 µL/band were applied on the HPTLC plates to obtain a final concentration range of 1–12 µg/band for the construction of a 7-point calibration curve. The HPTLC plates were developed using a mixture of methanol, n-hexane, and a 10:1 mixture of ethyl acetate:acetic acid in proportions determined by trial and error for each of the four bile salts (Table 1). The plates were developed at 25 ± 3 °C in an ADC2 automatic developing chamber (Camag^®^, Muttenz, Switzerland), and the solvent front was allowed to run to 60 mm from the lower edge of plate. The plates were dried for 5 min in a stream of cold air and dipped for derivatization in a p-anisaldehyde/sulfuric acid (anisaldehyde:acetic acid:methanol:sulfuric acid—1:20:170:10) solution at an immersion speed of 5 cm/s. The plates were allowed to dry for 30 s and oven-heated at 110 °C for 10 min so the colored bands could be seen. Plates were scanned and densitometrically evaluated with a TLC Scanner 4 (Camag^®^, Muttenz, Switzerland) monitored by WinCATS Planar Chromatography Manager v1.4.6 using a scanning slit of 4 × 0.30 mm^2^ at a scanning speed of 20 mm/s. The plates were scanned in absortion mode at 550 nm with a tungsten lamp. Peak profiles were generated from the captured images using the visionCATS v2.4 software (Camag^®^, Muttenz, Switzerland). The concentration of the samples was evaluated by interpolation in the 7-point absorbance vs. concentration calibration curve. 

### 2.4. Statistical Analysis

Data analysis was carried out with MyStat 12 software (SYSTAT Software, Inc., Chicago, IL, USA). The Shapiro–Wilk normality test, followed by a multiple comparison analysis (MCA), were used to detect significant differences between means at a significance level of α = 0.05. If the data follow a normal distribution, a parametric test such as one-way ANOVA with a post hoc test such as Tukey would be the MCA of choice. If this assumption could not be supported, a nonparametric test such as Wilcoxon would be used [17]. All data are presented as the arithmetic mean of three determinations ± standard deviation, unless stated otherwise.

## 3. Results and Discussion

### 3.1. BSH Activity Screening

The probiotic cultures were screened for BSH activity by qualitative direct plate assay. The results of this assay are shown in Table 2. It can be observed that BSH activity could be detected in all the probiotic strains with the exception of *S. boulardii*. The only strain that showed activity against the four conjugated bile acids was *L. plantarum* DGIA1. Three strains showed activity against three bile acids, and one of them (*L. rhamnosus* GG) showed only weak activity against sodium taurodeoxycholate. This last result was unexpected because the bile resistance and the presence of a *bsh* gene is well documented for *L. rhamnosus* GG [18]. The high bile tolerance (up to 1%) of *S. boulardii* has been previously reported, even though the actual mechanism is still unknown [19]. Based on the preliminary BSH activity screening results and on the bile tolerance information reported by other authors, all the probiotic strains were selected for quantitative BSH activity determination toward the four bile acids.

### 3.2. Quantitative HPTLC BSH Assay

**Figure 2:**
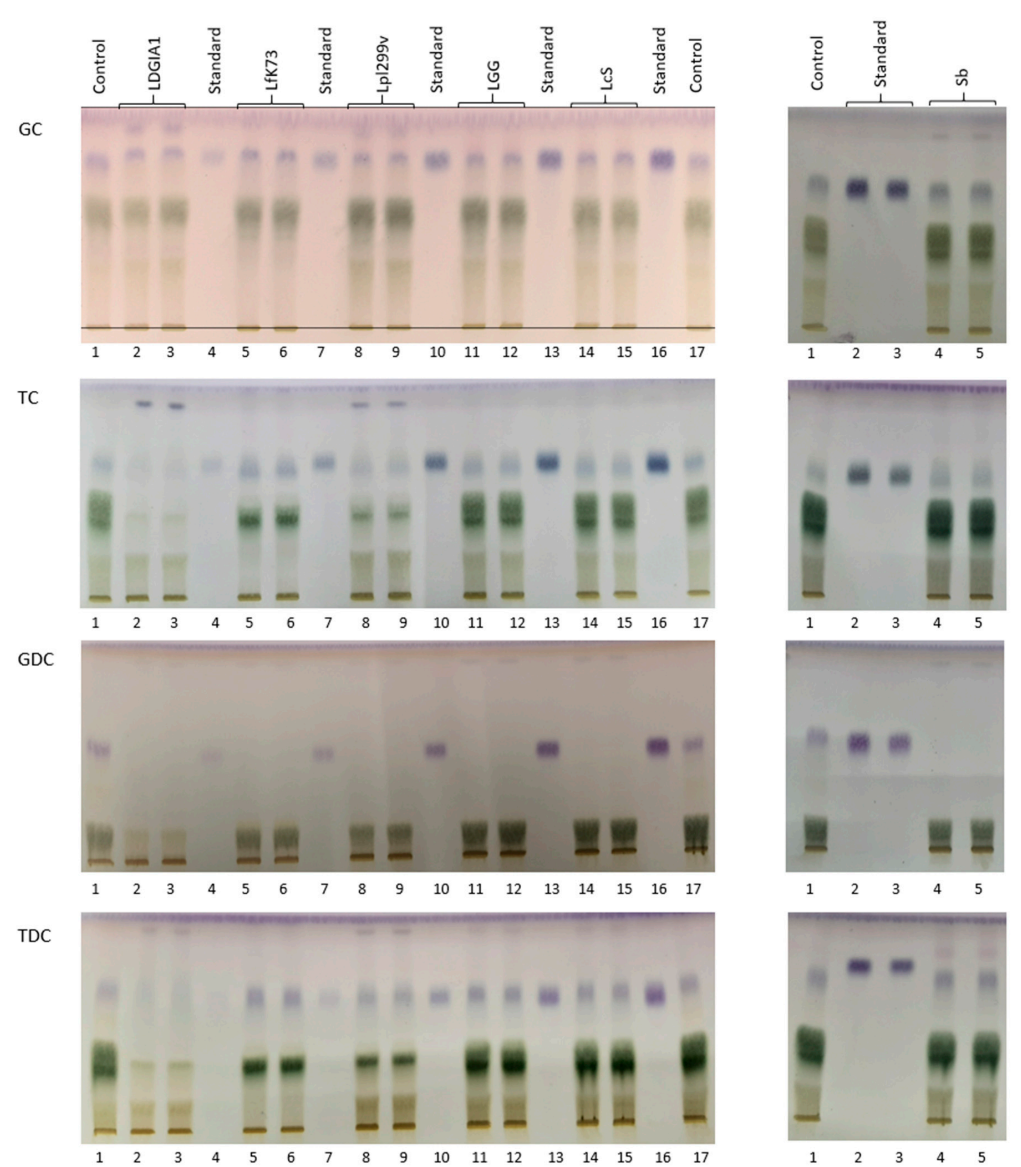
Quantitative assay of deconjugated bile acids in the cell-free supernatant from the six probiotic strains cultured at 37 °C for 24 h in MRS broth (YPD broth for SB) separated on HPTLC plates and derivatized with p-anisaldehyde/sulfuric acid. GC, sodium glycocholate; TC, sodium taurocholate; GDC, sodium glycodeoxycholate; TDC, sodium taurodeoxycholate; LDGIA1, *Lactobacillus plantarum* DGIA1; LfK73, *Lactobacillus fermentum* K73; Lpl299v, *Lactobacillus plantarum* 299v; LGG, *Lactobacillus rhamnosus* GG; LcS, *Lactobacillus casei* Shirota; SB, *Saccharomyces boulardii*.

Previous reports have shown that HPTLC is a rapid quantitative determination method for the deconjugation reaction of primary and secondary bile salts by probiotics [17]. The HPTLC plates after being run and developed are presented in Figure 2. The bile salt deconjugation activity by strains of probiotics is shown in Table 3. When the deconjugation activity data were assessed for normality, the Shapiro–Wilk test indicated a strong evidence for departure from normality (*p* < 0.05). In this case, the multiple comparison analysis testing was performed by the nonparametric Wilcoxon test using a significance level of α = 0.05 to detect differences among the deconjugation activities on the four bile salts for each strain. All six strains were able to completely deconjugate sodium glycodeoxycholate (100% hydrolysis). *L. plantarum* 299v had the highest specific BSH activity against sodium glycocholate (85% deconjugation) and *L. plantarum* DGIA1 had the highest specific BSH activity against sodium taurocholate and taurodeoxycholate (81% and 92% deconjugation, respectively). The highest overall deconjugation ability was observed in *L. plantarum* DGIA1, with values from 69 to 100% hydrolysis for the four bile salts, so it can be considered as the best BSH probiotic producer in this study.

### 3.3. Lactobacillus casei Shirota

*Lactobacillus casei* Shirota is a well-known probiotic and facultative heterofermentative LAB that has been given the Generally Recognized as Safe (GRAS) status by the FDA. *L. casei* Shirota provides health benefits derived from the regulation of the intestinal microbiota, improvement of abdominal dysfunction, prevention of infections, and modulation of the inflammatory and immune responses [20]. In general, *L. casei* strains have been extensively used in the dairy industry for centuries in the elaboration of cheeses and fermented dairy beverages. Additionally, many strains of *L. casei* confer general health benefits by acting as probiotics [21]. The reports of the presence of BSH in *L. casei* strains have been controversial. There are some studies where the authors have not found the activity of this enzyme in their *L. casei* strains [10,22]. However, there are others (including this study) in which good BSH activities have been detected [9,23,24,25]. The comparative values of the deconjugation percentage for sodium glycocholate and taurocholate can be observed in Table 4. All of the strains (including *L. casei* Shirota) showed a higher BSH activity in the case of the sodium glycocholate. According to Table 3, the preferred substrate for the BSH of *L. casei* Shirota is sodium glycodeoxycholate, although it can also have a weak action against sodium taurodeoxycholate. González-Vázquez et al. [26] reported that sodium taurocholate is the best substrate for the BSH of *L. casei* strains J57 and Shirota. 

### 3.4. Lactobacillus fermentum K73

*L. fermentum* K73 is a probiotic heterofermentative LAB isolated from Colombian suero costeño. It has the ability to absorb cholesterol and is reported to have good BSH activity [14]. There are many reports dealing with the presence of BSH in *L. fermentum* strains [9,22]. The substrate specificity varies with the strain. Cueto and Aragón [14] isolated three strains with good BSH activity (K11, K73, and K75) that were able to deconjugate sodium glycocholate. On the other hand, Jiang et al. [19] isolated two strains (ZL4 and L545) with activity against sodium taurocholate and taurodeoxycholate, but no activity against sodium glycocholate or glycodeoxycholate could be detected. Similar results were obtained by Moser and Savage [28] using the ATCC strains 11976 and 23271. In this study, the strain K73 showed a high activity against sodium glycocholate and glycodeoxycholate (61% and 100% deconjugation, respectively) and a relatively weak activity against sodium taurocholate and taurodeoxycholate (24% and 4% deconjugation, respectively). It is important to highlight that K73 is, to our knowledge, the only strain reported to have deconjugating activity against all four bile salts. 

### 3.5. Lactobacillus rhamnosus GG

*L. rhamnosus* GG is a well-known probiotic and facultative heterofermentative LAB. This microorganism has the ability to survive and thrive at gastric acid pH and in the presence of bile. In addition, it can adhere to enterocytes while inhibiting some pathogens and reducing inflammatory processes [29]. There are several studies reporting the presence of BSH in *L. rhamnosus* strains [9,30]. The presence of BSH in *L. rhamnosus* GG has been reported previously [18]. The reports on the BSH substrate specificity are scarce. Dong and Lee [31] indicated that the BSH of *L. rhamnosus* E9 had a preference for sodium glycocholate. Tsai et al. [6] reported that *L. rhamnosus* NBHK007 had good activity against sodium taurocholate. In this study, the strain GG showed a high activity against sodium glycocholate and glycodeoxycholate (63% and 100% deconjugation, respectively) and a relatively low activity against sodium taurocholate or taurodeoxycholate (24% and 1% deconjugation, respectively). This is the first time that information is being provided about the deconjugation percentages of different bile acids by the GG strain. 

### 3.6. Lactobacillus plantarum 299v and DGIA1

*L. plantarum* 299v is an LAB used extensively as a probiotic in several formulations. It shows a broad range of beneficial effects on human health, including the treatment of gastrointestinal and allergic diseases, obesity, metabolic syndrome, type 2 diabetes, nonalcoholic fatty liver, hypercholesterolemia, autoimmune disorders, inflammation, and different types of cancer. These effects have been confirmed by clinical studies [32]. BSH activity is related to the cholesterol-lowering potential of LAB, and its presence has been demonstrated in many strains of *L. plantarum*, including the strain 299v [9,33,34,35,36]. Several studies on BSH substrate specificity have shown substrate preference toward glycine-conjugated bile salts [10,12,37,38]. There are also a few reports indicating a preference for taurine-conjugated bile salts [28] and others indicating that for the strains Lp80 [12] and Lp-onlly, Lp-529, and Lp501 [22] the substrate preference is unclear since the strains have good activity in the two kinds of conjugated bile acids. Table 3 shows that in this study, the results for BSH activity in the strain 299v indicate a preference (as in most studies) for the glycine-conjugated bile salts (85% and 100% deconjugation for sodium glycocholate and glycodeoxycholate, respectively, compared to 65% and 31% deconjugation for sodium taurocholate and taurodeoxycholate, respectively). However, in the case of *L. plantarum* DGIA1, the deconjugation activities were high in all cases (69%, 100%, 81%, and 92% for sodium glycocholate, glycodeoxycholate, taurocholate, and taurodeoxycholate, respectively). This could indicate that the strain DGIA1 has a very potent BSH capable of deconjugating glycine- and taurine-containing primary and secondary bile salts. These are characteristics not previously found in strains of *L. plantarum*. This finding also confirms the fact that strains from food origin, such as DGIA1, can have BSH activities equal to or stronger than strains isolated from the intestines of humans. However, the fact that strains with this activity are isolated most often from the intestines or feces from mammals still stands [9]. The probiotic potential of the halotolerant strain DGIA1 has been evidenced previously [39]. 

### 3.7. Saccharomyces boulardii

The probiotic yeast *S. boulardii* has been prescribed to humans for almost 40 years for the prevention and treatment of gastrointestinal disorders with a predominant inflammatory component [40]. The yeast has been used mainly for prophylaxis and treatment of diarrheal diseases caused by the administration of antibiotics or in the case of bacterial infections caused by *Escherichia coli, Clostridioides difficile, Vibrio cholera*, and *Helicobacter pylori* [41]. However, the probiotic characteristics of the yeast also include the ability to tolerate the presence of bile salts at concentrations up to 1% [19,42] through a mechanism that is still unknown. One possible explanation for this tolerance could be the presence of a BSH. To our knowledge, the detection of this enzyme has not been reported previously. In this study, the deconjugation activities of *S. boulardii* were good in the cases of sodium glycodeoxycholate, taurocholate, and taurodeoxycholate (100%, 57%, and 63%, respectively). Weak deconjugative activity (5%) was observed in the case of sodium glycocholate. This BSH activity in the yeast could be an additional explanation for the observed anti-hypercholesterolemic effect of the yeast. This effect, reported initially by Girard et al. [43] in hamsters, has been also observed in adult humans [44]. The authors observed a decrease in remnant lipoproteins after daily supplementation with the probiotic yeast for 8 weeks. This is very important, because these remnant lipoproteins induce the development of fat deposits in the arteries. Briand et al. [45] observed that daily administration of *S. boulardii* for 2 to 3 weeks in hamsters fed a high-cholesterol diet significantly reduced the total plasma cholesterol compared to control animals, and also modified the gut microbiota composition of the rodent. These modifications of the microbiota were correlated to variations of lipidemic values. Therefore, the presence of BSH in the yeast could also be important to explain the lowering of the plasma cholesterol levels.

## 4. Conclusions

Tolerance to bile acids is often cited as an important probiotic characteristic. However, there are several mechanisms behind this attribute, many of which are very important, because they are also involved in the reduction of the blood cholesterol level of the host. This study confirms the finding that, in the case of probiotic *Lactobacillus*, the BSH content and substrate specificity depend strongly on the species and strain. The most promising result from this research was the finding that the cheese-isolated strain DGIA1 of *L. plantarum* was able to achieve high levels of deconjugation in sodium glycocholate, glycodeoxycholate, taurocholate, and taurodeoxycholate. In addition, the confirmation of the presence of BSH in *S. boulardii*, which had not previously been investigated, is an important result that must be explored further. Taking this into consideration, the daily consumption of a double cream cheese portion or *S. boulardii* supplements could be recommended to decrease serum cholesterol levels.

## Figures and Tables

**Table 1:** Composition of the optimized developing solvents for the determination of four bile salts by HPTLC.

Bile Salt	n-Hexane (%)	Methanol (%)	Ethyl Acetate:Acetic Acid (10:1) (%)	hRf
TC	20	40	40	78
TDC	15	35	50	70
GC	10	40	50	70
GDC	30	20	50	52

GC, sodium glycocholate; GDC, sodium glycodeoxycholate; TC, sodium taurocholate; TDC, sodium taurodeoxycholate; hRf, retardation factor (100 × Rf) where Rf can be defined as the ratio of the distance traveled by the spot to the distance traveled by the solvent front.

**Table 2:** BSH activity of probiotic strains grown on bile salt—MRS medium as manifested by the formation of a precipitation zone around the colony. The diameters of the precipitation zones are expressed in mm.

Probiotic	Sodium Glycocholate	Sodium Glycodeoxycholate	Sodium Taurocholate	Sodium Taurodeoxycholate
*Lb. plantarum* 299v	1	3	0	2
*Lb. rhamnosus* GG	0	0	0	1
*Lb.plantarum* DGIA1	3	3	2	2
*Lb.casei* Shirota	1	0	2	1
*Lb. fermentum* K73	1	0	3	2
*S. boulardii*	0	0	0	0

**Table 3:** Bile salt deconjugation activity (%) of the six strains of probiotics evaluated by HPTLC.

Strain	GC	GDC	TC	TDC
Control	0 ± 1.2	0 ± 2.1	0 ± 1.41	0 ± 2.3
LcS	49 ± 8.1 ^a^	100 ± 0 ^b^	41 ± 2.25 ^a^	18 ± 0.24 ^c^
LDGIA1	69 ± 2.2 ^a^	100 ± 0 ^b^	81 ± 0.20 ^c^	92 ± 6.45 ^d^
Lfk73	61 ± 1.1 ^a^	100 ± 0 ^b^	24 ± 5.20 ^c^	4 ± 2.25 ^d^
LGG	63 ± 3.8 ^a^	100 ± 0 ^b^	24 ± 7.31 ^c^	1 ± 1.22 ^d^
Lpl299	85 ± 5.2 ^a^	100 ± 0 ^b^	65 ± 7.26 ^c^	31 ± 2.54 ^d^
SB	5 ± 3.6 ^a^	100 ± 0 ^b^	57 ± 1.07 ^c^	63 ± 6.01 ^c^

LDGIA1, *Lactobacillus plantarum* DGIA1; LfK73, *Lactobacillus fermentum* K73; Lpl299v, *Lactobacillus plantarum* 299v; LGG, *Lactobacillus rhamnosus* GG; LcS, *Lactobacillus casei* Shirota; SB, *Saccharomyces boulardii*. Results are expressed as mean ± SD, n = 3. ^a–d^ Means within a row with different lowercase letters are significantly different (*p* ≤ 0.05). Uninoculated tubes were used as controls.

**Table 4:** Comparative values of the deconjugation percentage for sodium glycocholate and taurocholate in different strains of *Lactobacillus casei*.

*Lb. casei* Strain	GC	TC	Reference
ASCC 1520	26	20.7	[23]
ASCC 1521	79.5	53.8	[23]
ASCC 290	68.9	43.6	[25]
E5	100.0	66.7	[27]
N19	35.7	25.0	[27]
Shirota	49 ± 8.1	41 ± 2.25	This study

## Data Availability

All the data are included in the article.
